# The Peculiarities of *Metopolophium dirhodum* (Walk.) Population Formation Depending on Its Clonal and Morphotypic Organization during the Summer Period

**DOI:** 10.3390/insects14030271

**Published:** 2023-03-08

**Authors:** Elena Gandrabur, Anton Terentev, Alexander Fedotov, Dmitriy Emelyanov, Alla Vereshchagina

**Affiliations:** 1 All-Russian Institute of Plant Protection, 196608 Saint Petersburg, Russia; admin@vizr.spb.ru (A.T.);; 2 Advanced Digital Technologies, Peter the Great St. Petersburg Polytechnic University, 195251 Saint Petersburg, Russia; afedotov@spbstu.ru

**Keywords:** aphids, *Metopolophium dirhodum*, population size, population dispersal, clones, morphotypes, emigrants, apterous exules, alate exules

## Abstract

**Simple Summary:**

The features of aphid biology are determined by the clonal structure of their populations and polymorphism. The alternation of winter (arboreal) and summer (herbaceous) hosts in aphid species is accompanied by an annual change in the relative frequencies of clones and morphotypes within clones. Clones and morphotypes are tools to allow aphid populations to remain stable within the same genotypes or form more harmful biotypes. In both cases, it is important to study the relationships between these intrapopulation structures. For the cereal pest *Metopolophium dirhodum* (Walk.), population formation depends on the diversity of their clonal and morphotypic composition. The parameters of reproduction and settlement for the summer morphotypes (emigrants, apterous and alate exules) of 10 different *M. dirhodum* clones on wheat were determined. It was shown that the reproductive parameters of individual morphotypes varied significantly among the clones. Compared to apterous or alate exules, the reproduction of emigrants was significantly lower. The reproduction of apterous exules was strongly influenced by the generation time during the summer and annual changes in clonal composition. Alate exules and alatoid nymphs were produced only by apterous exules.

**Abstract:**

The ecological plasticity of aphid populations is determined by their clonal and morphotypic diversity. Clones will be successful when the development of their component morphotypes is optimized. The purpose of this work was to reveal the peculiarities of clonal composition and the developmental characteristics of different summer morphotypes for the rose-grass aphid, *Metopolophium dirhodum* (Walk.), which is an important host-alternating cereal pest and a useful model species. During the experiments, aphids were kept under ambient conditions on wheat seedlings at natural temperatures and humidity levels. An analysis of the reproduction of summer morphotypes and the resulting composition of offspring found that variation among the clones and morphotypes, as well as generational effects and an influence of sexual reproduction (and interactions between all factors) influenced the population structure of *M. dirhodum*. The reproduction of emigrants was less among the clones than that of the apterous or alate exules. The number of offspring produced by apterous exules differed throughout the growing season (generational effects) and between years, with different clones exhibiting different responses. There were dispersing aphids only among the offspring of apterous exules. These results can contribute to future advances in the forecasting and monitoring of aphid populations.

## 1. Introduction

The importance of cereals as agricultural crops is difficult to overestimate [1,2]. Therefore, it is essential to control wheat pests, especially aphids, which can cause yield losses of 40–60% [3,4,5,6,7,8,9]. The destructiveness of aphids is determined by their high rate of reproduction, ability to spread quickly, high ecological plasticity, and the toxicity of their extraintestinal secretions [3,10,11]. Aphids may also serve as a vector for viral and mycoplasmal pathogens [12,13,14], and fungal diseases, which develop on aphid honeydew, decrease the photosynthetic capabilities of the host plant [4,10,11,13,14]. However, it is difficult to manage aphid pests because insecticide-resistant forms (biotypes) frequently emerge in aphid populations; for example, there has been an increase in the frequency of several insecticide-resistant biotypes of *Myzus persicae* (Sulz.) over the last 7 years [15]. Ultimately, the ability of aphid clones to diversify and adapt affects the cost and effectiveness of pest control [16,17,18,19,20].

The order of appearance, combination, and separation of the life cycle functions among morphotypes within aphid clones has become the basis of their unique ability for phenotypical plasticity, specialization, and their long-term coevolutionary relationships with plants. For these same reasons, aphids are capable of exhibiting population outbursts in modern agricultural biocenoses [20,21,22,23,24], but, despite the large number of mathematical models on aphid population dynamics, these population dynamics are difficult to predict [25,26,27]. The complex life cycles of aphids can complicate monitoring and control efforts, but the development of parthenogenesis can be considered as one of the most influential factors in aphid life cycles, polymorphism (polyphenism), and trophic evolution [28,29,30,31]. Clones and morphotypes (individually, as well as through interactions) are known to shape (or are hypothesized to shape) the “intrapopulation structure” and overall abundance of aphid pests, but there is insufficient knowledge of the factors responsible for producing sharp fluctuations in pest abundance and changes in intrapopulation structure, including the relationships between clones and morphotypes [22,25,32].

The destructiveness of aphids is related to their evolutionary histories with their host plants [16,28]. For heteroecious aphids, the alternation between winter (arboreal) and summer (herbaceous) hosts allows them to reproduce asexually (as clones) on secondary hosts in summer before returning to their primary hosts for sexual reproduction in autumn and to lay overwintering eggs. Polymorphism (polyphenism) allows for the production of alate morphotypes that migrate to herbaceous plants in spring (emigrants), disperse between secondary hosts in summer (alate summer exules; the term “exule” refers to an aphid in the parthenogenetic generation on its secondary host), and bring the sexes together in autumn (males and gynoparae, reproducing oviparae) [4,32,33,34,35]. Genetic methods can be used to study the complexity of the aphid population structure, which provides information not only on the molecular genetic basis on the importance of aphid diversification, but also on the phenotypic diversity in the ontogeny of individual morphotypes and their relative frequencies in different clonal populations [4,36,37,38,39,40,41].

The rose-grass aphid, *M. dirhodum*, is an economically significant pest of grains [4,42]. It is a cosmopolitan species that utilizes roses (*Rosa* L.) as primary hosts and grasses (mainly cereals) as secondary hosts [43]. There is evidence that, when feeding on leaves, *M. dirhodum* causes less damage to plants compared to other types of cereal aphids [3]. However, due to its topical specificity, *M. dirhodum* feeds on the flag and other leaves of cereals from heading to grain ripening—the key stages for yield formation [44]. Leaves, especially the flag leaf, are significant sources of assimilates for mobilization in grain [45]. Feeding from the phloem, aphids intercept the assimilates and impair the nutrition of the ear. Thus, *M. dirhodum* can feed on cereals for a much longer amount of time than some other species, and it is also capable of mass reproduction, intraspecific differentiation, and the transmission of viral infections [11,42]; in some countries, *M. dirhodum* is classified as a traditional pest of cereals along with *Rhopalosiphum padi* (L.) and *Sitobion avenae* F. [8,41,46,47,48]. The purpose of this work was to reveal the peculiarities of *M. dirhodum* population size variability by analyzing the reproduction and dispersal of the summer morphotypes (emigrants, apterae and alate exules) of different clones over the growing season and across multiple years.

## 2. Materials and Methods

### 2.1. Aphid Life Cycle

The rose-grass aphid, *M. dirhodum*, is a heteroecious species. In spring, fundatrices hatch from winter eggs and begin reproducing parthenogenetically for several generations on their primary host (wild and cultivated plants of *Rosa* L. spp.). In this way, each fundatrix establishes a clone before the spring emigrants migrate to a secondary host plant (family Poaceae). On this secondary host, parthenogenetic reproduction continues for many generations throughout the summer, producing summer apterous and alate exules. In late summer, winged males and gynoparae appear in aphid colonies and migrate back to their primary hosts. The gynoparae give birth to sexual females, which mate with males and produce overwintering eggs [49,50,51].

### 2.2. Primary (Winter) Host

Three-year-old *R. canina* L. seedlings were used in the experiments as primary hosts. The seedlings were obtained in spring from the Komarov Botanical Institute of the Russian Academy of Sciences (BIN RAS) and were immediately transplanted into plastic vessels of a 35 cm diameter and 40 cm height. The plants (five seedlings, one per vessel) were grown under ambient conditions underneath an awning in a place protected from winds at natural temperatures and humidity levels, and their vegetation continued throughout the years of research.

### 2.3. Wheat (Summer Host)

The spring soft wheat *Triticum aestivum* L. var. *lutescens* cv. ‘Leningradskaya 6’ was used in the experiments as secondary hosts. The wheat was sown in ceramic vessels of an 18 cm diameter and 20 cm height. The plant density was 15 in each vessel. The vessels were kept under conditions similar to the previous.

### 2.4. Collection and Maintenance of Aphid Clones

The *M. dirhodum* clones used in the experiment were established from aphids collected in the vicinity of St. Petersburg, Leningrad Oblast, Russian Federation (59°56′19″ N, 30°18′50.8″ E). The aphids were collected from wheat crops in summer, and the clones were established from the aphids collected several kilometers apart; for each clone, one field-collected apterous female was placed in a vessel of wheat using a brush, and the vessel was placed in a wooden frame covered with a fine mesh. Colonies were maintained by transferring the aphids into new vessels with fresh plants every 20–25 days; infested leaves were removed from the source colony and placed into the new vessels, and the aphids moved to the new plants within two days. Throughout the summer, colonies were maintained on wheat under conditions that mimicked natural conditions (the temperatures and humidity levels were similar, but without the effects of resource limitation, entomophages, plant diseases, rain, or wind). In autumn, *M. dirhodum* migrating alates began to congregate on the fine mesh in the upper part of the cages, so the fine meshes (with alate) were placed in new vessels (also placed in a wooden frame covered with fine mesh) that contained five rose seedlings (one per vessel), where the aphids could lay their eggs (at the thorns) for overwintering. The following spring, two female fundatrices, on different shoots of each rose seedling, were allowed to produce offspring (the other fundatrices were removed with a brush); for each colony, the offspring of a total of 10 fundatrices were taken into account. The collection and maintenance of the aphid clones were carried out annually in a similar way.

### 2.5. Experiments

To study the formation of aphid populations, the reproductive rate and dispersal ability were measured as indicators of aphid development. These indicators were based on the counts of the number and morphotype composition of the offspring of one individual (in an isolated vessel with wheat) for the first 14 days of reproduction (P14) in four replications of each of the 10 clones. This is the period when the first alate females of the first daughter generation and apterous females of the second daughter generation mature (based on the data on the development of *R. padi*) and start reproducing [52]. The timing of counting the number of alate exules and alatoid nymphs (future alate) in each morphotype offspring made it possible to determine the ability of aphids to disperse.

The experiments were carried out in three stages.

The first stage, conducted in June 2020, investigated the effects of the morphotype (emigrant, apterous exule, and alate exule) and the clone (#1–10) on the reproduction and dispersal ability of aphids. Each emigrant used in this experiment was collected individually from the colonies on the rose, and the apterous and alate exules were collected individually as nymphs (which were produced by single mothers of each clone in isolated vessels to smooth out the transgenerational effects of crowding on the composition of the offspring) from the colonies on wheat.

The second stage, conducted in June–August 2020, investigated the effects of the clones on the reproduction and dispersal ability of apterous exules over the course of the growing season. The apterous individuals used in this experiment were prepared in the same manner as in the first experiment.

The third stage investigated the effects of a clone on the midsummer (July) reproduction and the dispersal ability of apterous exules over four consecutive years (2017–2020). The apterous individuals were prepared following the same methods as the second experiment.

The air temperatures during the experiments were measured using a Thomas Scientific labForce^®^ Thermometer (Table 1).

The statistical processing of the results, was made by a two-way analysis of variance utilizing Fisher’s test (F-test) and Tukey’s test using StatSoft^®^ STATISTICA 12 software. The source data can be downloaded from the publishers cite (see Supplementary Materials).

## 3. Results

### 3.1. The Effects of Morphotypes and Clones on the Reproduction and Dispersal Ability of M. dirhodum (June 2020)

There were significant differences in the number and composition of the offspring of each of the three morphotypes at the beginning of the season (Table 2, June). All of the morphotypes were evaluated simultaneously; at this time, the emigration was still ongoing, but the first colonies of the emigrant offspring on the cereals had already appeared.

As a result of the two-way analysis of variance, it was shown that the formation of the number of *M. dirhodum* was significantly influenced by the characteristics of a clone (set of morphotypes) and even more by the characteristics of the morphotypes (set of clones), as well as the interaction of both factors (Table 3).

In this period, the P14 index was significantly lower for the emigrants (63 ± 7 individuals) than for the apterous exules (119 ± 12 individuals) and lower than for alate exules (123 ± 13 individuals). There were no significant differences between the apterous and alate exules in this index. The significance of the differences was determined using Tukey’s test for the “morphotype” factor in the two-way AOV (Table 2, June; Table 4).

We also noted differences in the morphotype composition of the offspring. Alate exules, as well as alatoid nymphs, were only found in the colonies formed by apterous exules (Table 2).

### 3.2. The Effects of a Clone on the Reproduction/Dispersal Ability of M. dirhodum Apterous Summer Exules over the Course of the Growing Season (June, July, August 2020)

The number of offspring produced by apterous exules changed throughout the summer period: it was 32.0% in June and 60.0% of the offspring maximum in August (Table 2). The number of the offspring varied significantly among clones, and there was also a significant link between a clone and a month (which suggests that the seasonal population dynamics vary from one clone to another) (Table 5).

At the same time, the percentage of alate and alatoid nymphs in the offspring decreased noticeably in July, despite the higher level of reproduction by exules, and both the alate and alatoid nymphs were absent in August (Table 2).

### 3.3. The Effects of Clones on the Midsummer (July) Reproduction/Dispersal Ability of M. Dirhodum Apterous Exules over Four Consecutive Years (2017–2020)

It was revealed that the difference between the years of the experiment had a significant influence on the number and composition of apterous summer exules’ offspring in July. The number of apterae exules’ offspring turned out to be maximal in 2020 and minimal in 2019 (22.8% of the maximum) (Table 2—July; Table 6).

The number of apterous summer exules also varied depending on a clone as well as the interaction between a clone and a year (Table 7). Additionally, the ability to produce alate and alatoid nymphs in *M. dirhodum* had turned out to be low, with a maximum in 2017 (Table 2 July).

## 4. Discussion

Clones that possess a set of adaptations optimized for all morphotypes will be more successful and will have a greater impact on structuring a pest population and driving its dynamics [53], but this phenomenon is not yet understood well enough to create models that reliably predict the population dynamics of aphids in agricultural ecosystems [26]. Because many studies only utilize laboratory populations without accounting for the natural variations in temperature, humidity, and illumination, there is a need to conduct experiments that better simulate natural conditions for a better characterization of the influence that genotype-phenotype relationships have in the life cycles of aphids [3,21,38,40,54,55]. The present study addresses this knowledge gap by investigating the features of the clonal composition and development of *M. dirhodum* morphotypes under semi-natural conditions in Northeastern Europe/Northwestern Russia.

Compared to *R. padi*, the reproductive potential and migratory abilities of *M. dirhodum* are significantly lower under similar climatic conditions [56,57,58]. While emigrants of certain *R. padi* clones can occasionally exhibit unusually high reproductive rates (with a single female producing up to 2000 individuals or more in the first 14 days of reproduction [52,59,60], the studied clones of *M. dirhodum* did not reach such astronomical reproductive rates. In 2020, the emigrants of all *M. dirhodum* clones produced an average of only 63 ± 7 offspring, yet this species is still capable of exceeding the threshold at which pest control measures are required (10 aphids on a stem at 50% plant infestation) [61]. Emigrants may have a reduced fecundity as the season progresses; by the end of the migration period, the individual rate of reproduction decreases due to an increase in the colony density and a decrease in the nutritional quality of the host plant [60,62]. Interestingly, the emigrants of *M. dirhodum* did not produce alate or alatoid offspring; for *R. padi*, we previously found that the percentage of alatoid nymphs varied from 0.6% to 27.4% and the number of alate from 0 to 5.7%, depending on the clone [60]. In 2017, the apterous summer exules of *R. padi* were each producing more than 600 offspring [60], whereas those of *M. dirhodum* were producing approximately 360 offspring. This relatively lower level of reproduction has been noted by other authors under different climatic conditions as well [56,63]. The morphotype composition of the offspring also differed this year; *R. padi* produced a greater percent of dispersing morphotypes (alate and alatoid nymphs, in total) than *M. dirhodum*, up to 26.5% of the offspring [64] compared to 7.82% (Table 6, 2017). Many factors influence polyphenism in aphids [57,64]; in the present study, the role of aphid clones and morphotypes is discussed.

The observed differences in the reproductive rates of various morphotypes and clones of *M. dirhodum* may have been the result of basic genetic differences or differential responses to environmental conditions. Because most of the variations in the P14 index were explained by morphotypes than by clones (set of morphotypes) (Table 3), this suggests that phenotypes (morphotypes) are more plastic than genotypes (clones) and will be the first to respond to changes in environment conditions, although the range, direction, and magnitude of this response can be expected to vary among clones (clone–morphotype interactions). Thus, both morphotype and clone are relevant factors to consider when monitoring pest populations and developing predictive models. Additionally, since the reproductive rates of apterous exules changed throughout the season (peaking in July), despite the fact that there were no notable changes in temperatures throughout the summer and that the variety and developmental phase of the host plant were held constant, this suggests that other eco-temporal variables may be important to consider as well; other research teams have established similar patterns of annual changes in the dates of peak abundance of *M. dirhodum* in the field [47,65,66]. Finally, because reproductive rates varied with clones across the years—despite there being no correlation in the reproductive rates of apterous exules and the average monthly temperatures over the four-year study period—this suggests that the cycling of parthenogenetic generations and sexual reproduction might affect the reproductive rates of subsequent generations. In previous studies, we noted annual changes in the development and fecundity of the individuals of this species that were correlated with temperature and humidity [47,49]. Altogether, this highlights the stochastic effects of the interannual variability in seasonal temperatures on *M. dirhodum* and further study on this subject is warranted to better understand the implications for pest management.

The study of phenotypic plasticity is fundamental in understanding the interactions between aphids and in the context of environmental factors [21]. In the present study, the greatest differences in the size and composition of aphid populations arose due to annual differences in clonal organization. There is no doubt that high fecundity and the optimal morphotype ratio in a population provides a great advantage during periods of high intraspecific competition; however, with increasing habitat heterogeneity (moving away from monocultures), many different clones are more likely to coexist, which may also reduce the rate of microevolutionary processes. We also found that the relative performance of different clones can essentially reverse in summer due to morphotype-specific developmental parameters, even though the nutritional environment remained unchanged [59], although these movements appear to be difficult to predict. Other factors may affect the relative success of different clones and their component morphotypes as well, such as the relative abundance of alate migrants, the emergence of adaptive mutations, and the relationships with entomophages, parasites, endosymbionts, and mutualists [4,58,67]. Ultimately, the clonal nature of aphid populations greatly affects their ecology and evolution in various important ways, but it is not yet fully understood [58].

Because of the possibility that a clone with a high reproductive rate will emerge, the clonal variability and intraclonal composition of aphid populations need to be monitored regularly. Due to the high adaptability of aphid populations, farmers should work with local scientific groups to conduct genetic monitoring to identify resistant populations in the field. Aside from this, standard IPM practices are still recommended, including closely monitoring crops for pest damage, changing the crop variety (for improved mechanisms of plant defense), and limiting chemical treatments to avoid the emergence of insecticide-resistant aphid populations.

The results from the present study offer potential for future research in the monitoring and forecasting of aphid populations, with important applications in creating models that can be used to improve pest management.

## 5. Conclusions

The size of the *M. dirhodum* population that forms on secondary hosts depends on the reproduction and offspring composition of summer ontogenetic morphotypes (emigrants, apterous and alate exules) within the complex of its constituent clones. In addition to the influences of clones and morphotypes, the time within and between growing seasons also affected the population dynamics of *M. dirhodum*; generational effects are a component of the changes within a single growing season, and the effects of sexual reproduction contribute to variation from one year to the next. The observed differences in reproduction were more influenced by the traits of individual morphotypes than by clonal specificity (complex of morphotype) within each year. However, the changes in the offspring number produced by apterous summer exules was largely influenced by annual variability among the clones and by the timing of the generations, and only apterous exules produced a progeny capable of dispersal. The present study of the roles of phenotypic changes in the population formation of *M. dirhodum* are of special importance due to the ever-evolving pest status of aphids, as well as in our broader understanding of the dynamics of agroecological systems.

## Figures and Tables

**Table 1:** The characteristics of the air temperatures during the research period.

Year	Month (Data)	Temperature (Average Values)		
		Max.	Min.	All Period
2017	July (1–24)	18.1	12.0	15.1
2018	July (1–24)	21.8	14.4	18.1
2019	July (1–24)	18.3	11.6	15.0
2020	July (1–24)	21.7	12.9	17.3
2020	June (8–31)	21.7	12.9	17.3
	July (1–24)	19.4	12.6	16.0
	August (1–24)	20.9	13.0	17.0

**Table 2:** The number and composition of summer morphotypes offspring of various clones and generations of *Metopolophium dirhodum* (Walk.) in 2020.

Clone	June					July			August
	Emigrants **	Apterous Exules			Alate Exules **	Apterous Exules			Apterous Exules
	P14 *	P14	Offspring Composition, %		P14	P14	Offspring Composition, %		P14
			Alate Exules	Alatoid Nymphs			Alate Exules	Alatoid Nymphs	
1	57 ± 5	83 ± 16	3.6	1.2	160 ± 17	294 ± 18	0	0	230 ± 25
2	38 ± 11	105 ± 10	8.6	0.9	137 ± 20	440 ± 26	0.2	0	214 ± 20
3	100 ± 12	180 ± 10	3.3	4.4	136 ± 7	300 ± 22	2.3	0	274 ± 29
4	46 ± 9	120 ± 19	3.3	2.5	86 ± 9	435 ± 25	0	0	219 ± 36
5	74 ± 15	151 ± 12	2.6	9.3	146 ± 28	312 ± 16	0.3	0	351 ± 30
6	56 ± 10	138 ± 9	5.1	3.6	195 ± 18	259 ± 32	0.4	0	184 ± 34
7	83 ± 11	155 ± 13	1.3	2.6	76 ± 11	565 ± 17	0	0	131 ± 19
8	59 ± 8	88 ± 9	1.1	1.1	97 ± 14	371 ± 32	0.8	0.5	178 ± 22
9	35 ± 6	111 ± 10	0.9	3.6	65 ± 10	421 ± 30	0.2	0	269 ± 25
10	82 ± 16	57 ± 11	0	0	127 ± 9	319 ± 39	0	0.6	167 ± 22
X ± SE	63 ± 7	119 ± 12	3.0 ± 0.8	3.0 ± 0.8	123 ± 13	372 ± 28	0.4 ± 0.2	0.1 ± 0.1	222 ± 20

Notes: * P14 (X ± SE)—the number of the offspring of one female after 14 days of initial reproduction; ** there were no dispersing individuals in the offspring of the morphotype.

**Table 3:** The results of the two-way variant analysis (F-test) of the influences of the clones and summer morphotypes on the number of *Metopolophium dirhodum* (Walk.) (June 2020).

Factor	Effect				
	Df	SS	MS	F	*p*
Clone	9	55,238	6138	8.9	<0.001
Morphotype	2	89,117	44,559	64.8	<0.001
Clone-morphotype	18	72,482	4027	5.9	<0.001

Note: Df—the degrees of freedom; SS—the sum of squares; MS—the mean sum of squares due to the source; F—F-statistic (Fisher’s test); *p*—*p*-value.

**Table 4:** (**a**). The results of the two-way analysis of variance (F-test) of the influences of a clone and summer morphotype on the number of *Metopolophium dirhodum* (Walk.) (June 2020) (Table 3) for the morphotype factor. (**b**). The differences (Tukey’s test, Q) among the summer morphotypes offspring (P14) of *Metopolophium dirhodum* (Walk.) (June 2020).

**(a)**					
**Source of Variation**	Effect *				
	**Df**	**SS**	**MS**	**F**	***p***
Between groups	2	89,117	44,559	64.8	<0.001
Within groups	90	61,886	688	64.8	<0.001
Total	92	151,003		64.8	<0.001
**(b)**					
**Comparison**	**Absolute Mean Difference**		**Q 0.01 (Critical Value)**		**Significant**
Emigrants vs. apterous exules	53		31.2		Yes
Apterous exules vs. alate exules	4		31.2		No
Emigrants vs. alate exules	60		31.2		Yes

Note: * see Table 3.

**Table 5:** The results of the two-way variant analysis (F-test) of the influences of clones and months (generation) on the number of apterae *Metopolophium dirhodum* (Walk.) (June, July, August 2020).

Factor	Effect *				
	SS	Df	MS	F	*p*
Clone	143,555	9	15,951	7.6	<0.001
Morphotypeotype	1,292,709	2	646,355	306.6	<0.001
Clone-morphotypeotype	367,643	18	20,425	9.7	<0.001

Note: * see Table 3.

**Table 6:** The number and composition of apterous exules offspring of *Metopolophium dirhodum* (Walk.) clones in July 2017–2019 *.

Clone	2017			2018			2019 **
	Offspring Composition, %			Offspring Composition, %			P14
	P14	Alate Exules	Alatoid Larvae	P14	Alate Exules	Alatoid Larvae	
1	194 ± 17	7.2	5.2	286 ± 24	0	1.4	164 ± 15
2	298 ± 24	0	0	479 ± 40	0.6	1.9	33 ± 2
3	349 ± 26	4.3	3.7	390 ± 27	0	1.5	34 ± 2
4	264 ± 22	3.4	5.7	369 ± 24	0	0	146 ± 10
5	139 ± 10	10.8	13.7	320 ± 21	0	1.3	50 ± 7
6	273 ± 7	2.2	5.5	345 ± 31	0	0	104 ± 13
7	337 ± 27	2.1	1.2	318 ± 28	0	0.8	56 ± 9
8	127 ± 11	3.9	1.6	232 ± 19	0	0	67 ± 8
9	293 ± 35	0.7	1.5	186 ± 24	0	0	155 ± 18
10	360 ± 21	2.8	3.1	533 ± 40	0	0	39 ± 3
X ± SE	263 ± 25	3.7 ± 0.1	4.1 ± 1.2	346 ± 31.6		0.7 ± 0.2	85 ± 16

Note: * 2020—see Table 2 (July); ** there were no dispersing individuals in the offspring of the apterous exules in 2019.

**Table 7:** The results of the two-way variant analysis (F-test) of the influences of clones and years on the number of apterous *Metopolophium dirhodum* (Walk.) (July 2017–2020).

Factor	Effect *				
	SS	Df	MS	F	*p*
Clone	290,581	9	32,287	14.9	<0.001
Year	2,013,809	3	671,270	310.8	<0.001
Clone-year	771,390	27	28,570	13.2	<0.001

Note: * see Table 3.

## Data Availability

Data are contained within the article or Supplementary Material.

## Supplementary Materials

The following supporting information can be downloaded at: https://www.mdpi.com/article/10.3390/insects14030271/s1, Data are contained within the article or Supplementary Material.
